# Thermal Weight Determination and Interstate Coupling in State-Averaged ADAPT-VQE

**DOI:** 10.1021/acs.jctc.5c01313

**Published:** 2025-12-04

**Authors:** Harper R. Grimsley, Francesco A. Evangelista

**Affiliations:** Department of Chemistry and Cherry Emerson Center for Scientific Computation, 1371 Emory University, Atlanta, Georgia 30322, United States

## Abstract

Characterizing electronic thermal states at low temperatures is an important but challenging task in quantum chemistry and condensed matter physics, making it a prime candidate for a useful application in quantum computing. One of the most successful methods for state preparation on quantum computers is the Adaptive, Problem-Tailored (ADAPT) Variational Quantum Eigensolver (VQE), which has recently been generalized to treat excited states within a state-averaged framework as well as Gibbs states. In this work, we introduce Helmholtz-Optimized Thermal (HOT) ADAPT-VQE, an ancilla-free strategy for preparing Gibbs states that directly minimizes the Helmholtz free energy by targeting the dominant eigenstates of the thermal ensemble. We demonstrate the usefulness of HOT-ADAPT-VQE by predicting the free energy of two model systems with strongly correlated ground states: (1) the Fe^2+^ cation in a magnetic field and (2) a [Cu_2_O_7_]^10–^ fragment of the Mott insulator La_2_CuO_4_. Our results demonstrate that HOT-ADAPT-VQE significantly improves upon Gibbs-state estimates from multistate variants of ADAPT-VQE, often with substantially shallower quantum circuits, making it a promising candidate for thermal-state calculations.

## Graphical abstract

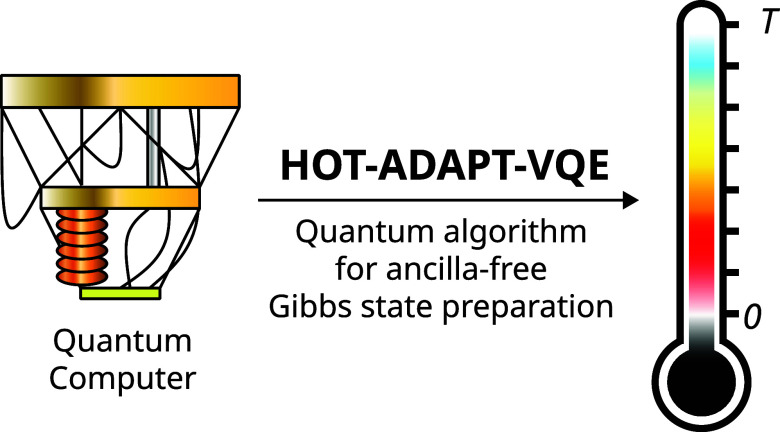

## 1 Introduction

One of the most important applications of quantum chemistry is characterizing the ground- and excited-state electronic structures of molecules. While the description of individual states of chemical systems is important, chemical processes occur at finite temperatures, making it necessary in many situations to consider thermal ensembles. Understanding the effect of temperature on molecular systems is crucial in various applications, including the design of materials, the prediction of chemical reactions, and the interpretation of molecular spectra. Finite-temperature extensions of many classical methods have been proposed, including matrix product state methods,^1–3^ quantum Monte Carlo,^4–6^ dynamical mean-field theory,^7,8^ density functional theory,^9,10^ perturbation theory,^11–13^ coupled cluster theory,^14–16^ nonperturbative thermal cluster cumulant methods,^17–20^ and configuration interaction methods.^21,22^ However, the characterization of finite-temperature chemical systems with classical computers faces several challenges. Thermal states are largely difficult to predict for the same reasons as for individual excited states: They may display strong electron correlation, and the Hilbert or Fock space of all determinants is combinatorially large.^23^ This difficult, relevant classical problem motivates a quantum solution.

Quantum chemistry has long been pursued as a potential application of quantum computers. Twenty years ago, the quantum phase estimation (QPE) algorithm^24^ was applied in the context of electronic structure theory.^25^ Since then, substantial efforts have been directed toward the design of algorithms for the prediction of ground and excited electronic states of molecules.^26–37^ While less studied, various quantum methods exist for predicting thermal states. These usually follow one of two strategies: (1) variational minimization of a cost function^38,39^ or (2) sampling states obtained with quantum simulation of imaginary time evolution.^31,40–42^ Unfortunately, these methods are plagued by various issues such as deep circuits, ancilla qubit requirements, and difficulties in performing exact entropy measurements.

The first issue, circuit depth, is shared by all quantum algorithms during the ongoing “noisy, intermediate-scale quantum” (NISQ)^43^ era. Deep quantum circuits correspond to lengthy time evolutions of the quantum registers. The longer the evolution, the more difficult it is to avoid decoherence of the qubits or buildup of gate errors. An early quantum algorithm proposed to address this problem was the variational quantum eigensolver (VQE).^26^ In VQE, a correlated state is generated from a reference state |ϕ_0_⟩ via a unitary operator *Û*(**θ**) that depends on a set of classical, real-valued parameters **θ**. The energy of the state $Û\left(\theta \right)|\phi_{0}\rangle$ is given as

$$E\left[\theta \right]=\left\langle \phi_{0}\left|{Û}^{†}\left(\theta \right)ĤÛ\left(\theta \right)\right|\phi_{0}\right\rangle$$ 1

where *Ĥ* is the Hamiltonian of the system. In VQEs, *Û*(**θ**) is designed such that it is a relatively short circuit, and |ϕ_0_⟩ is easily prepared on a quantum register. These choices make measurements of *E*[**θ**] tractable on the NISQ hardware. The energy and wave function of the system are approximated by variationally minimizing *E*[**θ**] with respect to **θ**, using a classical optimization algorithm to update **θ** and the quantum computer to measure *E*[**θ**] and optionally its gradient with respect to the parameters **θ**.

The VQE algorithm is not uniquely defined due to the freedom in the choice of *Û*(**θ**). It was originally proposed to use the unitary (*U*) version of the coupled cluster singles and doubles (CCSD) ansatz^44^

$$Û\left(\theta \right)=\exp\left(\underset{ia}{\sum }\theta_{i}^{a}\left({â}_{i}^{a}-{â}_{a}^{i}\right)+\underset{\begin{array}{l} i<j\\ a<b \end{array}}{\sum }\theta_{ij}^{ab}\left({â}_{ij}^{ab}-{â}_{ab}^{ij}\right)\right)$$ 2

where the indices *i*, *j* (*a*, *b*) correspond to occupied (empty) spin-orbitals of the reference state. The operators ${â}_{i}^{a}={â}_{a}^{†}{â}_{i}$ and ${â}_{ij}^{ab}={â}_{a}^{†}{â}_{b}^{†}{â}_{j}{â}_{i}$ are single and double Fermionic excitations, respectively, expressed in terms of creation ( ${â}_{p}^{†}$ ) and annihilation ( ${â}_{q}$ ) operators. Since it is impractical to implement the UCCSD unitary on quantum computers, a factorized (disentangled) form of UCCSD is employed instead. This disentangled form of UCC is an independent ansatz that can be systematically improved by adding higher-order excitations^45^ and with an operator ordering dependence that leads to different variational solutions and corresponding energies.^46^

In practice, many of the operators in the factorized UCCSD ansatz will frequently have little bearing on the energy accuracy attainable with the ansatz. Including superfluous operators increases the circuit depth, making standard factorized UCCSD inefficient.

The adaptive, problem-tailored (ADAPT-) VQE was introduced to address these issues for Fermionic ansätze.^47^ The details of this algorithm will be addressed in Section 2, but the general idea is to start with a pool of simple anti-Hermitian generators $\left\{{Â}_{\mu }\right\}$ , corresponding to primitive unitaries $\left\{\exp\left(\theta_{\mu }{Â}_{\mu }\right)\right\}$ . The ansatz *Û*(**θ**) is iteratively constructed from these candidate unitaries based on greedy, first-order estimates of their ability to decrease the energy.

To obtain multiple states at once, the ansatz *Û*(**θ**) could be used to form a set of orthogonal many-body states by choosing a set of orthonormal references {|ϕ_*i*_⟩} and using the same unitary to form the set of correlated states $\left\{Û\left(\theta \right)|\phi_{i}\rangle \right\}$ . Orthonormalization is ensured by the fact that for any pair of states, $\langle \phi_{i}|{Û}^{†}\left(\theta \right)Û\left(\theta \right)|\phi_{j}\rangle =\left\langle \phi_{i}|\phi_{j}\right\rangle =\delta_{ij}$ . Diagonalizing *Ĥ* in the basis of orthonormal states of this form can be used to obtain excited states in quantum methods. Moreover, state-averaged VQE methods exist, which optimize **θ** to minimize the average energy of a set of orthogonal states, such as the subspace-search (SS-)^28^ and multistate, contracted (MC−)^29^ VQE methods. VQEs based on a multideterminantal reference state have also been considered^33,48–53^ and, more recently, ADAPT-VQE has also been generalized to use a state-averaged framework.^54,55^ In several state-averaged VQE methods, the user must define a set of reference states and specify the relative importance of the states by choosing a weight for the contribution of each state to the averaged energy. This formalism will be described more explicitly in Section 2 in the context of ADAPT-VQE. While the optimal choice of weights in state-averaged methods is a subject of ongoing research,^56,57^ the state-averaged ADAPT-VQE methods do not currently have a black box way of determining the weights of each state. The same open problem^58–60^ exists in traditional state-averaged complete-active-space self-consistent-field methods (SA-CASSCF),^61^ which simultaneously optimize the orbital basis and determinant coefficients of a multideterminantal state.

ADAPT-VQE was first extended to thermal state preparation in the ADAPT-VQE-Gibbs method.^39,62^ This approach minimizes a proxy function for the Helmholtz free energy of a mixed state (ensemble) at a fixed temperature. Through purification, the mixed state is obtained as a partial trace of a pure state defined in a larger qubit space, necessitating the use of ancilla qubits. While the proxy function does not require entropy measurements, it still cannot be efficiently measured without approximation. Approximate estimates of the proxy objective function cause its minimum to no longer exactly correspond to that of the Helmholtz free energy.

In this work, we propose a different ADAPT-VQE strategy for thermal states that requires no ancilla, avoids problematic entropy measurements, and pursues the exact minimum of the Helmholtz free energy. As we will discuss in Section 2, rather than attempting to optimize a general mixed state in the 2^*N*^ Hilbert space represented by *N* qubits, we assume the special case that there are only a small number *k* of important references, which is realistic for low temperatures or systems with large excitation gaps. In making this assumption, we can design a unitary specifically to optimize the most important states in a physically informed way. We denote our method the Helmholtz-Objective Thermal ADAPT-VQE method (HOT-ADAPT-VQE). During preparation of this manuscript, Sambasivam et al.^63^ introduced the Truncated Eigenvalue Parameterized Initial Density (TEPID-) ADAPT-VQE method, which, like our method, directly targets the Helmholtz free energy. While our proposed algorithm is similar to TEPID-ADAPT-VQE, it requires no ancilla qubits. As detailed in Section 2, HOT-ADAPT-VQE is also distinct in the use of a specific decoupling strategy. We explain in Section 2 how this decoupling allows us to reduce a naïvely *k* ^2^-term density operator representation of the Gibbs state to only *k* terms without sacrificing accuracy. Finally, in this work, we extend the domain of application of thermal states beyond model Hamiltonians, focusing on ab initio Hamiltonians of two prototypical 3d transition metal systems: (1) the Fe^2+^ cation and (2) a cluster model of cuprates. We discuss the differences in the algorithms more extensively in Section 2.

## 2 Theory

### 2.1 State-Averaged ADAPT-VQE

The most intuitive way to understand HOT-ADAPT-VQE is to begin from comparable multistate, temperature-agnostic alternatives. Two algorithms have been proposed to generalize the traditional ADAPT-VQE algorithm^47^ to a multistate formalism that simultaneously optimizes a set of states. The first method is the state-averaged (SA-) ADAPT-VQE-SCF.^54^ SA-ADAPT-VQE-SCF adaptively optimizes the molecular orbitals, as well as the ansatz itself. Freezing the orbitals gives the simplified Multistate-Objective, Ritz-Eigenspectral (MORE-) ADAPT-VQE algorithm introduced by the authors.^55^ For simplicity, we restrict our focus to MORE-ADAPT-VQE, ignoring the added benefits of orbital optimization.

The objective function to be minimized in MORE-ADAPT-VQE is the state-averaged energy (*E*_MORE_)­

3 $$E_{MORE}\left[\theta \right]=\sum_{i}^{k}w_{i}\langle \phi_{i}|{Û}^{†}\left(\theta \right)ĤÛ\left(\theta \right)|\phi_{i}\rangle$$

where ${\left\{w_{i}\right\}}_{i=1}^{k}$ is a set of *k* user-defined, positive weights which sum to 1, and ${\left\{|\phi_{i}\rangle \right\}}_{i=1}^{k}$ is a set of *k* user-specified, orthonormal references, typically chosen to be Slater determinants. We note in passing that one could consider the MORE-ADAPT-VQE objective function as the energy expectation value of an ensemble of states

4 $$E_{MORE}\left[\theta \right]=Tr\left({\mathrm{\rho ̂}}_{MORE}\left[\theta \right]Ĥ\right)$$

where ${\mathrm{\rho ̂}}_{MORE}\left[\theta \right]$ is a density matrix expressed in terms of state weights, reference states, and the unitary *Û*(**θ**) as

5 $${\mathrm{\rho ̂}}_{MORE}\left[\theta \right]=\sum_{i}^{k}w_{i}Û\left(\theta \right)|\phi_{i}\rangle \langle \phi_{i}|{Û}^{†}\left(\theta \right)$$

To construct *Û*(**θ**), the user must specify a pool of anti-Hermitian generators $\left\{{Â}_{\mu }\right\}$ , which is usually chosen to be universal for a pure state. In this work, we always use the universal pool of anti-Hermitian generalized single and double^64^ excitations

6 $$\left\{{Â}_{\mu }\right\}=\left\{{â}_{p}^{q}-{â}_{q}^{p}\right\}\cup \left\{{â}_{pq}^{rs}-{â}_{rs}^{pq}\right\}$$

where the indices *p*, *q*, *r*, and *s* run over all spin-orbitals.

An ansatz *Û*(**θ**), parameterized by a vector **θ** of real numbers, is initialized to the trivial identity operator

7 $${Û}^{\left(0\right)}\left(\theta \right)=1̂$$

To choose our first element from the pool, we consider each operator ${Â}_{\mu }$ as a candidate. For each candidate operator, we construct the candidate energy functional

8 $$E_{candidate\left(SA\right)}^{\mu }\left[\theta^{\prime }\right]=\sum_{i}^{k}w_{i}\left\langle \phi_{i}\left|{Û}^{†}\left(\theta \right)e^{-\theta^{\prime }{Â}_{\mu }}Ĥe^{\theta^{\prime }{Â}_{\mu }}Û\left(\theta \right)\right|\phi_{i}\right\rangle$$

where θ′ is a new, undefined parameter. For each candidate energy, the gradient is computed with respect to the new parameter at θ′ = 0, giving a first-order estimate of the importance of the associated generator

9 $$g_{\mu }=\frac{\partial E_{candidate\left(SA\right)}^{\mu }\left[\theta^{\prime }\right]}{\partial \theta^{\prime }}{|}_{\theta^{\prime }=0}=\sum_{i}^{k}w_{i}\left\langle \phi_{i}\left|{Û}^{†}\left(\theta \right)\left[Ĥ,{Â}_{\mu }\right]Û\left(\theta \right)\right|\phi_{i}\right\rangle$$

The operator ${Â}_{\mu }$ with the largest absolute gradient |*g*_μ_| is then appended to the left side of *Û*(**θ**)­

10 $${Û}^{\left(1\right)}\left(\theta \right)=e^{\theta_{1}{Â}^{\left(1\right)}}1̂$$

where θ_1_ has been appended to **θ** and ${Â}^{\left(1\right)}={Â}_{\mu }$ .

At this point, the state-averaged energy *E*_MORE_[**θ**] (see eq 3) is optimized with a nonadaptive VQE, using $Û\left(\theta \right)={Û}^{\left(1\right)}\left(\theta \right)$ as the ansatz.

After the VQE subroutine update of **θ**, another unitary operator is chosen based on its candidate gradient and appended to *Û*(**θ**). This process is repeated iteratively until some user-defined convergence criteria are achieved. One choice is to terminate the algorithm at some fixed number of operators in the ansatz. This is a natural choice in situations in which the main hardware limitation is an inability to implement the deeper circuits required for more operators. Another common choice for ADAPT-VQE methods is to terminate the algorithm when the norm (typically the *L* ^2^ or *L* ^∞^ norm) of the candidate gradients {*g*_μ_} becomes sufficiently low. This choice is a more appropriate representation of situations where measurement error is the primary difficulty such that gradient resolution is the limiting factor of the hardware.

Once the convergence criteria are reached, a Ritz diagonalization of the Hamiltonian is performed in the basis of correlated reference states ${\left\{Û\left(\theta \right)|\phi_{i}\rangle \right\}}_{i=1}^{k}$ , yielding *k* eigenstates {|Ψ_*i*_⟩} and energies {*E*_*i*_}. The corresponding eigenstates are given by

11 $$|\Psi_{i}\rangle =\sum_{j}^{k}\;Û\left(\theta \right)|\phi_{j}\rangle C_{ji}$$

where **C** is a *k* × *k* matrix of expansion coefficients. Due to the state-averaged formalism, *Û*(**θ**) is the same for all references. This allows the diagonalization to be written in terms of **C** and a diagonal matrix of the eigenenergies (**E**)­

12 H̅C=CE

where the subspace Hamiltonian matrix **H̅** has elements

13 $${\mathrm{H̅}}_{ij}=\langle \phi_{i}|{Û}^{†}\left(\theta \right)ĤÛ\left(\theta \right)|\phi_{j}\rangle$$

The construction of **H̅** does not require ancilla qubits since off-diagonal elements ${\mathrm{H̅}}_{ij}$ can be obtained by performing measurements on the state $\frac{1}{\sqrt{2}}Û\left(\theta \right)\left(|\phi_{i}\rangle +|\phi_{j}\rangle \right)$ as discussed in refs 28 and 29 . Note that when *k* = 1, there is only one reference, and MORE-ADAPT-VQE simplifies to a traditional ADAPT-VQE calculation.

Both SA-ADAPT-VQE-SCF and MORE-ADAPT-VQE rely on weights chosen by the user. The optimal choice of weights is a complicated and open problem,^56,57^ so a method that determines the weights for the user is an attractive feature. This partially motivates the HOT-ADAPT-VQE algorithm along with the desired application of predicting thermal states.

### 2.2 HOT-ADAPT-VQE

To generalize MORE-ADAPT-VQE to a system at a finite temperature, we begin by defining a variational energy functional whose minimum corresponds to a thermal state. For a system of *N* electrons at temperature *T*, the canonical ensemble Gibbs state ρ̂ minimizes the Helmholtz free energy F , given by

14 $$F=U-k_{B}TS$$

where *k*_B_ is the Boltzmann constant. The internal energy *U*, not to be confused with the unitary operator *Û*(**θ**) which defines the ansatz, is the Hamiltonian expectation value, written in terms of Hamiltonian eigenstates {|Ψ_*i*_⟩} and corresponding probabilities {*p*_*i*_}­

15 $$U=Tr\left(\mathrm{\rho ̂}Ĥ\right)=\sum_{i}^{\left|H\right|}p_{i}\left\langle \Psi_{i}\left|Ĥ\right|\Psi_{i}\right\rangle$$

where |H| indicates the dimension of the Hilbert space H . The von Neumann entropy *S* is given as

16 $$S=-Tr\left(\mathrm{\rho ̂}\log\mathrm{\rho ̂}\right)=-\sum_{i}p_{i}\;\log p_{i}$$

The probability for the system to occupy the microstate |Ψ_*i*_⟩ follows from minimizing the free energy and is given by the Boltzmann distribution

17 $$p_{i}=\frac{1}{Z}e^{-\beta \left(E_{i}-E_{0}\right)}$$

Here, we have introduced the inverse temperature, β = 1/*k*_B_ *T*, and the canonical partition function

18 $$Z=\sum_{i}e^{-\beta \left(E_{i}-E_{0}\right)}$$

which ensures that the probabilities sum to one.

MORE-ADAPT-VQE generalizes quite naturally to the prediction of thermal states due to its connection through the density matrix representation to a weighted statistical ensemble of states. In eq 5, we have already seen that one can use ADAPT-VQE to minimize the ensemble energy. To extend MORE-ADAPT-VQE to a thermal state, we approximate the canonical Gibbs state as

19 $$\mathrm{\rho ̂}\approx {\mathrm{\rho ̂}}_{HOT}=\sum_{i}^{k}p_{i}|\Psi_{i}\rangle \langle \Psi_{i}|$$

This form of ${\mathrm{\rho ̂}}_{HOT}$ contains two assumptions. The first one is to restrict the sum over eigenstates to a set of *k* low-energy states ${\left\{|\Psi_{i}\rangle \right\}}_{i=1}^{k}$ . For low temperatures, most of the eigenstates of the Hamiltonian with *E*_*i*_ ≫ *E*_0_ are not significantly populated and can be neglected. This assumption is also used in TEPID-ADAPT-VQE. The second assumption is that the *k* excited states ${\left\{|\Psi_{i}\rangle \right\}}_{i=1}^{k}$ can be represented, as in eq 11, as linear combinations of correlated references ${\left\{Û\left(\theta \right)|\phi_{i}\rangle \right\}}_{i=1}^{k}$ for an adaptively constructed ansatz *Û*(**θ**), where expansion coefficients **C** are obtained by solving eq 12. The HOT-ADAPT-VQE expression for ${\mathrm{\rho ̂}}_{HOT}$ generalizes the expression of ${\mathrm{\rho ̂}}_{MORE}$ (see eq 5) by replacing the reference states |ϕ_*i*_⟩ with the set of multideterminantal states $\left\{\sum_{j=1}^{k}|\phi_{j}\rangle C_{ji}\right\}$ . The additional flexibility of the latter ansatz is expected to lead to more accurate results for small values of *k*. As pointed out in ref 52, it is difficult to design circuits that implement arbitrary multideterminantal references of the form $\sum_{j=1}^{k}|\phi_{j}\rangle C_{ji}$ . A significant advantage of the HOT-ADAPT-VQE method is that it does not require preparing complicated multireference states on a quantum register, as demonstrated later in this section. Note that HOT-ADAPT-VQE is guaranteed to converge to the exact finite temperature density matrix in the limit of k→|H| since in this limit the basis of states $\left\{|\Psi_{i}\rangle =\sum_{j=1}^{\left|H\right|}\;Û\left(\theta \right)|\phi_{j}\rangle C_{ji}\right\}$ can be exact eigenstates, irrespective of *Û*(**θ**) due to the availability of a sufficient number of parameters in the expansion coefficient matrix, albeit with exponential scaling. However, as discussed below, there are several scenarios where accurate thermal properties can be obtained with only a small number of low-energy states (small *k*), such as low-temperature regimes, systems with large excitation gaps, or impurity problems, where thermal effects are localized to a small subsystem.

Introducing the definition of ${\mathrm{\rho ̂}}_{HOT}$ into the Helmholtz free energy functional leads to the following expression for F in terms of the ensemble probabilities ({*p*_*i*_}), expansion coefficients ({*C*_*ji*_}), and unitary [*Û*(**θ**)]

20 $$F\left[Û\left(\theta \right)\right]=\sum_{ijl}^{k}p_{i}C_{ji}C_{li}^{*}\left\langle \phi_{l}\left|{Û}^{†}\left(\theta \right)ĤÛ\left(\theta \right)\right|\phi_{j}\right\rangle +\frac{1}{\beta }\sum_{i}p_{i}\;\log p_{i}$$

While we have omitted their dependence on **θ** from the equations, the ensemble weights and expansion coefficients appearing in HOT-ADAPT-VQE are not truly independent variational parameters since, given *Û*(**θ**), they can be computed noniteratively from eqs 17 and 12, respectively. Throughout this work, we also assume that the orbitals are kept frozen. Therefore, all variational conditions involve the parameters in the HOT-ADAPT-VQE ansatz, the Boltzmann probabilities, and the expansion coefficients. The first term on the r.h.s. of eq 20 requires the measurement of *k* ^2^ elements of the subspace Hamiltonian **H̅**. As mentioned in the previous subsection, this can be accomplished without ancilla qubits using the method of ref 34. The second term (entropy) requires no additional quantum measurements to compute since the probabilities {*p*_*i*_} can be determined by classically diagonalizing **H̅** and using the eigenvalues to predict the Boltzmann distribution. Having an efficient way to measure F[Û(θ)] , we now have what we need to build an ancilla-free finite-temperature VQE. For a general, fixed choice of the structure of *Û*(**θ**), we denote the process HOT-VQE and implement it in a one-step fashion, where {*p*_*i*_} and **C** are assigned their optimal values based on **θ** for any given free energy or gradient calculation according to eqs 12 and 17.

To create an adaptive variant of HOT-VQE, our last task is to identify a criterion for choosing which operator to add to the unitary. Following the approach used in TEPID-ADAPT-VQE, it is natural to use the single-parameter gradients of F in analogy with eq 9. We begin by turning eq 20 into a candidate energy functional for operator ${Â}_{\mu }$

21 $$F_{candidate}^{\mu }\left[\theta^{\prime }\right]=\sum_{ijl}^{k}p_{i}C_{ji}C_{li}^{*}\left\langle \phi_{l}\left|{Û}^{†}\left(\theta \right)e^{-\theta^{\prime }{Â}_{\mu }}Ĥe^{\theta^{\prime }{Â}_{\mu }}Û\left(\theta \right)\right|\phi_{j}\right\rangle +\frac{1}{\beta }\sum_{i}p_{i}\;\log p_{i}$$

By always keeping F optimized (within the constraints of normalization and ∑_*i*_ *p*_*i*_ = 1) with respect to {*p*_*i*_} and **C**, its gradient is greatly simplified. Because the optimal ensemble weights and CI coefficients correspond to a variational solution, F must have vanishing first derivatives with respect to both {*p*_*i*_} and **C**. These conditions correspond to {*p*_*i*_} satisfying the Boltzmann distribution and **C** diagonalizing the subspace Hamiltonian. In particular, we note that the stationarity of F with respect to {*p*_*i*_} implies that there is no contribution from the entropy in gradients, so that while F≠U , we still have $\frac{\partial F}{\partial \theta }=\frac{\partial U}{\partial \theta }$ . This is actually a troubling feature of this free energy functional despite the simplified gradient. As we will discuss in Section 4, this can manifest in the form of premature convergence due to the depopulation of excited states. Following these considerations, the gradient expression simplifies to

22 $$\begin{array}{ll} g_{\mu } & ={\frac{\partial F_{candidate}^{\mu }\left[\theta^{\prime }\right]}{\partial \theta^{\prime }}|}_{\theta^{\prime }=0}\\ & =\sum_{ijl}p_{i}C_{ji}C_{li}^{*}\left\langle \phi_{l}\left|{Û}^{†}\left(\theta \right)\left[Ĥ,{Â}_{\mu }\right]Û\left(\theta \right)\right|\phi_{j}\right\rangle \end{array}$$

Choosing the operator ${Â}_{\mu }$ with the largest $\left|g_{\mu }\right|$ gives us our prescription for ansatz construction. The overall algorithm is shown graphically in Figure 1 and in pseudocode in Alg. 1. Note that since in HOT-ADAPT the Boltzmann probabilities are generally unequal, the energy expression cannot be simplified by using the trace invariance property as done in MORE-ADAPT.

**Figure 1:**
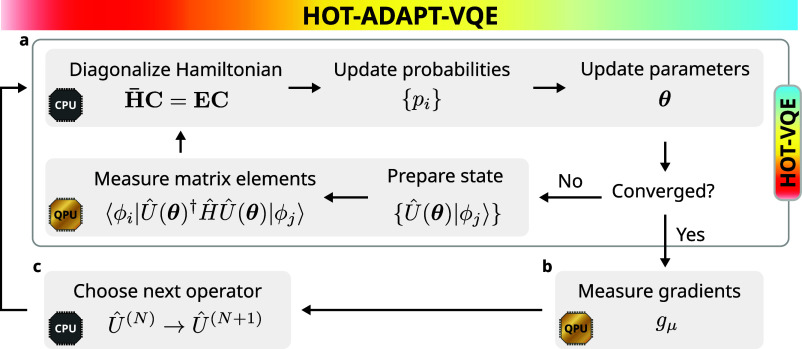
HOT-ADAPT-VQE procedure. (a) In the HOT-VQE subroutine, the CPU (central processing unit) uses classical optimization techniques to minimize the free energy F with respect to the coefficient matrix **C**, ensemble weights {*p*_*i*_}, and parameters **θ**, for a fixed unitary ansatz ${Û}^{\left(k\right)}\left(\theta \right)$ containing a product of *k* unitaries. The QPU (quantum processing unit) is responsible for preparing states and measuring the Hamiltonian matrix elements and gradients with respect to the parameters. (b) Once the HOT-VQE-VQE subroutine is converged, the QPU is used to measure the free energy gradient (*g*_μ_) for all candidate operators. (c) The next operator is chosen to maximize the absolute gradient (|*g*_μ_|) and added to ${Û}^{\left(k\right)}\left(\theta \right)$ , altering the ansatz to ${Û}^{\left(k+1\right)}\left(\theta \right)$ . The procedure continues by entering the HOT-ADAPT-VQE subroutine to optimize the updated ansatz. The algorithm is terminated when all user-specified criteria are met.

For an ensemble ${\mathrm{\rho ̂}}_{HOT}$ of the form in eq 19, we have described how to obtain the individual energies $\left\{\langle \Psi_{i}\left|Ĥ\right|\Psi_{i}\rangle \right\}$ , from which the quantities *U*, *S*, and F can all be obtained through trivial classical calculations. The ensemble expectation value of any other observable *Ô* can be computed in the same fashion

23 $$Tr\left({\mathrm{\rho ̂}}_{HOT}Ô\right)=\sum_{i}^{k}p_{i}\left\langle \Psi_{i}\left|Ô\right|\Psi_{i}\right\rangle$$

where {|Ψ_*i*_⟩} is the set of eigenstates of *Ĥ*.

We briefly remark on the energy additivity of HOT-ADAPT-VQE for noninteracting fragments represented by product states (size consistency). Consider the case where the Hamiltonian for a system *A*, ${Ĥ}_{A}$ , has a well-converged HOT-ADAPT-VQE solution

24 $${\mathrm{\rho ̂}}_{HOT}^{A}=\sum_{i}^{k}p_{i}^{A}|\Psi_{i}^{A}\rangle \langle \Psi_{i}^{A}|$$

where |Ψ_*i*_^A^⟩ is the exact *i*th lowest-energy eigenstate of ${Ĥ}_{A}$ . As a simple example, let us consider the situation where all the energies are degenerate with the value *E*_A_. It follows that all the ensemble weights {*p*_*i*_^A^} will be equal to $\frac{1}{k}$ and the corresponding free energy will be equal to $F_{A}$ . Suppose a noninteracting copy of A is introduced (which we refer to as B). In that case, the corresponding Hamiltonian will be $Ĥ={Ĥ}_{A}+{Ĥ}_{B}$ and the free energy should be equal to $2F_{A}$ with the ensemble given by the product state ${\mathrm{\rho ̂}}_{HOT}^{A}⊗{\mathrm{\rho ̂}}_{HOT}^{B}$ , with ${\mathrm{\rho ̂}}_{HOT}^{B}={\mathrm{\rho ̂}}_{HOT}^{A}$ . Let us consider the free energy for the case where we include only some number of eigenstates *k*′ for the tensor product Hilbert space $H_{S}=H_{A}⊗H_{B}$ . The value of *k*′ corresponds to the total number of eigenstates being used to describe a mixed state in the enlarged Hilbert space, not the number of additional eigenstates relative to *k*. Because the energy of each state for each subsystem is *E*_A_, and there is no coupling between the two subspaces, the energy of each eigenstate in the product space is 2*E*_A_. Additionally, since the eigenstates within the product space are all degenerate, the probabilities will be uniform in a Boltzmann distribution, and because we are only using *k*′ eigenstates, the probability of each one in our ensemble will be $p_{i}=\frac{1}{k^{\prime }}$ . Plugging into the definition of the Helmholtz free energy, we have a supersystem energy $F_{S}$ , given as

25 $$F_{S}=\sum_{i}^{k^{\prime }}\frac{1}{k^{\prime }}2E+\frac{1}{\beta }\sum_{i}^{k^{\prime }}\frac{1}{k^{\prime }}\log\frac{1}{k^{\prime }}$$

26 $$=2E+\frac{1}{\beta }\log\frac{1}{k^{\prime }}$$

27 $$=2E+2\frac{1}{\beta }\sum_{i}^{k}\frac{1}{k}\log\frac{1}{k}-2\frac{1}{\beta }\sum_{i}^{k}\frac{1}{k}\log\frac{1}{k}+\frac{1}{\beta }\log\frac{1}{k^{\prime }}$$

28 $$=2F_{A}-2\frac{1}{\beta }\log\frac{1}{k}+\frac{1}{\beta }\log\frac{1}{k^{\prime }}$$

29 $$=2F_{A}+\frac{1}{\beta }\left(2\;\log k-\log k^{\prime }\right)$$

30 $$=2F_{A}+\frac{1}{\beta }\log\frac{k^{2}}{k^{\prime }}$$

Note that we reintroduced the original *k* in order to describe the supersystem free energy $F_{S}$ in terms of the subsystem free energy and the number of eigenstates *k* used for the subsystem. It follows that HOT-ADAPT-VQE is not generally size-consistent unless the second term in the final free energy expression, $\log\frac{k^{2}}{k^{\prime }}$ is null, a condition met when the number of supersystem references scales quadratically with system size (*k*′ = *k* ^2^).

To illustrate this point, a simple toy example can be constructed by considering an H_2_ molecule in the STO-3G basis with a bond distance of 1 Å and computing the lowest 4 FCI energies. Using these four states to form an optimized Gibbs state gives a free energy of $F_{H_{2}}=-5.229E_{h}$ at *T* = 1 × 10^6^ K. It follows that for a Hamiltonian which is a tensor product of two isolated H_2_ molecules (i.e., the two are infinitely separated), the free energy should be −10.457 *E*_h_ for size consistency to be satisfied. Using only the 8 lowest-energy products of H_2_ eigenstates, however, gives a free energy of −8.437 *E*_h_. To obtain the full −10.457 *E*_h_, 16 (4^2^) lowest-energy product states must be used. It is worth noting that replacing any of the 16 lowest-energy product states with a state that is higher in energy also fails to give the −10.457 *E*_h_ energy. This exemplifies that to satisfy energy additivity, it is necessary to simultaneously satisfy the condition *k*′ = *k* ^2^ and include the correct *k* ^2^ states.

It should be noted that the previous situation assumes the worst-case scenario of a temperature sufficiently high that all eigenstates are relevant in both subsystems. Another more realistic situation is that of two coupled subsystems with very different energy scales, with the best case scenario being that at temperature *T*, subsystem A has *k* significantly populated eigenstates ${\left\{|\Psi_{i}^{A}\rangle \right\}}_{i=1}^{k}$ , while subsystem B has only one low-lying eigenstate |Ψ_0_^B^⟩. This scenario captures impurity problems in physics and chemistry, such as a high-spin transition metal surrounded by solvent molecules, where the first subsystem has far more relevant states than the second. In this situation, there will be only *k* significantly populated tensor product states $\left\{{|\Psi_{i}\rangle }_{A}⊗{|\Psi_{0}\rangle }_{B}\right\}$ in the ensemble, such that *k* does not need to be increased at all to capture the Gibbs state of the supersystem. One could repeat this process, adding an additional copy of the second subsystem, e.g., adding another solvent molecule, but by the same reasoning, this would not increase the number of relevant tensor product states. This implies that as long as the thermal effects are localized on the original subsystem, *k* does not have to increase with the system size for HOT-ADAPT-VQE to prepare the exact ensemble, making the method relevant for a general class of impurity problems.

A toy example illustrating this idea is to consider a stretched H_2_ molecule with an interatomic distance of 5 Å (subsystem *A*) and a weakly correlated H_2_ molecule with an interatomic distance of 1 Å (subsystem *B*) at 1000 K, using a minimal basis (STO-3G). Computing the free energy from the lowest four exact eigenstates (*k* = 4), the results for the two individual subsystems are −0.938 and −1.101 *E*_h_, respectively. Note that for the 1 Å geometry, this is just the ground-state energy since the energy levels are too far apart for excited states to be significantly populated. For the noninteracting composite system, an expansion based on the lowest four product states (built from the exact eigenstates) yields a free energy of −2.039 *E*_h_, accurately recovering the exact sum of the free energies of the isolated subsystems.

A major difference between HOT- and TEPID-ADAPT-VQE is that the latter does not address the coupling between the correlated reference states $\left\{Û\left(\theta \right)|\phi_{i}\rangle \right\}$ . That is, in analogy to the MORE-ADAPT-VQE method, it expresses its density matrix ( ${\mathrm{\rho ̂}}_{TEPID}$ ) as a sum of projectors onto the individual VQE states $\left\{Û\left(\theta \right)|\phi_{i}\rangle \right\}$

31 $${\mathrm{\rho ̂}}_{TEPID}=\sum_{i}p_{i}Û\left(\theta \right)|\phi_{i}\rangle \langle \phi_{i}|{Û}^{†}\left(\theta \right)$$

HOT-ADAPT-VQE, however, uses projectors onto diagonalized states {|Ψ_*i*_⟩} to form its density matrix ${\mathrm{\rho ̂}}_{HOT}$ . This means that the HOT-ADAPT-VQE ensemble comprises states which are already decoupled through **H̅**, giving a potentially lower free energy for the same ADAPT-VQE ansatz *Û*(**θ**). The significance of this difference between the algorithms becomes apparent in the limit of *T* = 0. TEPID-ADAPT-VQE becomes equivalent to ADAPT-VQE, barring situations in which *Û*(**θ**) changes the energy orderings of the states $\left\{Û\left(\theta \right)|\phi_{i}\rangle \right\}$ as it is constructed. HOT-ADAPT-VQE, on the other hand, can still make use of its extra references by replacing |ϕ_0_⟩ with a continuously optimized linear combination of all of the references, giving a more flexible ansatz, even at *T* = 0. One can more generally think about the flexibility of the two ansätze by considering the requirements to obtain an arbitrary *k*-term density matrix

32 $$\mathrm{\rho ̂}=\sum_{i}^{k}p_{i}|\Psi_{i}\rangle \langle \Psi_{i}|$$

For TEPID-ADAPT-VQE to exactly describe this density matrix, it would have to obtain individual states $Û\left(\theta \right)|\phi_{i}\rangle$ such that

33 $$\left\{Û\left(\theta \right)|\phi_{i}\rangle \right\}=\left\{|\Psi_{i}\rangle \right\}$$

for all *i* < *k*. On the other hand, HOT-ADAPT-VQE requires only that

34 $$span\left\{Û\left(\theta \right)|\phi_{i}\rangle \right\}=span\left\{|\Psi_{i}\rangle \right\}$$

Because the *k* weights {*p*_*i*_} are not generally equal, the Helmholtz free energy is not trace-invariant, so that one will obtain different energies and gradients if the states $\left\{Û\left(\theta \right)|\phi_{i}\rangle \right\}$ are rotated among themselves.

To show this numerically, we calculated the operator addition gradients from HOT-ADAPT-VQE, with and without coupling, for a linear H_4_ molecule at 1000 K. A uniform spacing of 2 Å was used between each pair of adjacent hydrogen atoms. Canonical orbitals were used, and three references were chosen: the Aufbau determinant and the singly excited HOMO–LUMO determinants (|ϕ_0_⟩, |ϕ_1_^2^⟩, and $|\phi_{1̅}^{2̅}\rangle$ ). Using the same Fermionic pool used later in this work, HOT-ADAPT-VQE chose the operator ${â}_{00̅}^{33̅}-{â}_{33̅}^{00̅}$ with gradient 0.27 *E*_h_. Disabling the coupling, i.e., forcing **C** to be the identify matrix, led the algorithm to choose the operator ${â}_{11̅}^{22̅}-{â}_{22̅}^{11̅}$ with gradient 0.25 *E*_h_. In the coupled case, the formation of the spin-adapted triplet configuration state function (CSF) allowed HOT-ADAPT-VQE to identify a different operator than it would have otherwise chosen.

## 3 Computational Details

Single-particle states (orbitals) used to represent many-body states are obtained either from Hartree–Fock computations performed with the Psi4 software package^65^ or from complete-active-space self-consistent-field computations performed with the Forte plugin.^66^ In ref 8, we provide JSON (JavaScript Object Notation) files containing the value of the scalar energy (*V*), one-electron integrals *h*_*pq*_, and the antisymmetrized two-electron integrals ⟨*pq*∥*rs*⟩ = ⟨*pq*|*rs*⟩ – ⟨*pq*|*sr*⟩ for the two systems considered in this work. These quantities are given on a spin-orbital basis, ordered in such a way that the spin-up and spin-down functions for the *p*th spatial function correspond to even (2*p*) and odd (2*p* + 1) indices. All quantum algorithms were simulated in the absence of noise using a local modification of the QForte package.^67^ Simulations also did not include error due to finite sampling. We removed these error sources in order to consider the performance of the algorithm itself. We defer a more involved exploration of these topics to future work. This code is made available through local forks of the main Forte and QForte repositories (see the Code Availability statement). In modeling molecular systems at finite temperatures, we partition the orbital basis into three sets: core (doubly occupied), active (with variable occupation), and virtual (unoccupied). The exact eigenstates and corresponding eigenenergies were obtained via complete-active-space configuration interaction (CASCI), whereby we impose occupation restrictions consistent with our orbital partitioning to generate the full Hilbert space of *N*-electron determinants. All CASCI computations use the corresponding ground-state orbitals optimized at the restricted open-shell Hartree–Fock (ROHF) or state-specific CASSCF levels. All exact thermodynamic quantities were then computed by ensemble averaging, as shown in eq 17. For each system considered, we report the values of temperature (*T*), internal energy (*U*), entropy (*S*), free energy ( F ), and the average value of the square of the total spin operator ( ${Ŝ}^{2}$ ) in CSV (comma-separated values) files in ref 8.

In this work, the performance of HOT-ADAPT-VQE is compared to that of a finite-temperature variant of MORE-ADAPT-VQE. In the latter, we first optimize *Û*(**θ**) using equal weights for the *k* chosen references and then predict ensemble averages using a Boltzmann distribution of the decoupled eigenstates {|Ψ_*i*_⟩} according to eq 17. Reported ensemble properties are then computed according to eqs 14–16 and 23. Note that because the same individual states are being used to form the MORE-ADAPT-VQE ensembles at different temperatures, only one MORE-ADAPT-VQE calculation needs to be performed for a given system. In the formal limit of an exact ansatz, HOT-ADAPT-VQE and MORE-ADAPT-VQE would yield the same thermal state. The advantage of HOT-ADAPT-VQE is therefore not in the exact limit but in converging faster, that is, producing accurate finite-temperature results with significantly shallower circuits by directly targeting the Helmholtz free energy.

Reported critical temperatures are computed as

35 $$T_{c}=\frac{1}{k_{B}}\left(E_{N}-E_{0}\right)$$

where *E*_0_ is the energy of the ground state and *E*_N_ is the energy of an excited state, with respective ensemble weights *p*_0_ and *p*_N_. Through algebraic manipulation of eq 35, we see that we can define the critical temperature to be such that *p*_0_ is greater than *p*_N_ by a factor of *e*.

We discuss the choice of references for each molecular system in its respective subsection, but in both cases, we choose determinants rather than CSFs for the sake of simplicity, particularly given the high multiplicities involved. As mentioned previously, the anti-Hermitian generalized single and double (GSD) operator pool was used for all MORE- and HOT-ADAPT-VQE calculations. The Broyden–Fletcher–Goldfarb–Shanno (BFGS) algorithm was used for parameter optimization in all VQE subroutines.^68^ As our primary goal is to model finite-temperature effects, the systems considered in this work were modeled without the inclusion of an environment or counterion effects since the quantitative accuracy of our models is less important than the accuracy of HOT- and MORE-ADAPT-VQE in obtaining solutions for those models.

## 4 Numerical Simulations

### 4.1 Iron Cation

As our first illustrative example, we consider a model of the low-lying electronic states of the Fe^2+^ cation. This system was chosen due to its simplicity and the variety of spin states, making it an excellent benchmark for investigating spin-dependent thermal population effects. An active space consisting of six electrons in six orbitals (6e, 6o) was used, comprising the Fe 3d and 4s atomic orbitals from an ROHF computation in the cc-pVDZ basis.^69,70^ In our ROHF computation, we target the ground ^5^D state, optimizing a determinant with quantum numbers *S* = 2 and *M*_S_ = 2 and orbital occupation ${3d}_{z^{2}}^{2}$ ${3d}_{x^{2}-y^{2}}^{1}{3d}_{xy}^{1}{3d}_{yz}^{1}{3d}_{xz}^{1}$ . Due to the double occupancy of the 3d_*z* ^2^_ orbital, the ROHF solution breaks spherical symmetry, yielding nondegenerate orbital levels. This splitting is analogous to the presence of a weak crystal field or weak orbital interactions in a metal complex. These orbitals, together with their associated Hartree–Fock energies (in *E*_h_), are depicted in Figure 2. To weakly split the degenerate quintet ground state, we include a magnetic field aligned with the *z*-axis in the effective Hamiltonian

36 $${Ĥ}_{z}=Ĥ-1\times 10^{-4}{Ŝ}_{z}$$

The CASCI (exact diagonalization) energies of the lowest 25 states of the effective Hamiltonian are shown in Figure 2. Due to symmetry breaking, the 25-fold degenerate ^5^D ground state of Fe^2+^ artificially splits into five quintet states that span an energy range of ca. 260 meV (with the fourth and fifth groups being nearly degenerate). The field induces a 2.72 meV (100 μ*E*_h_) splitting within each group of the five components of the quintet state. Focusing on the thermodynamics of the lowest five states, this splitting corresponds to a critical temperature *T*_c_ = 31.6 K. To describe the Gibbs state within the range 0.5 ≤ *T* < 5 *T*_c_, we consider as references for MORE- and HOT-ADAPT-VQE a set of 16 determinants (enumerated in Table 1) in which the 3d_*z* ^2^_ orbital is doubly occupied and the other 3d orbitals are singly occupied. These references span values of *M*_S_ ranging from −2 to 2. The energy errors for MORE- and HOT-ADAPT-VQE are compared in Figure 3 for temperatures between 0.5 and 5 *T*_c_.

**Figure 2:**
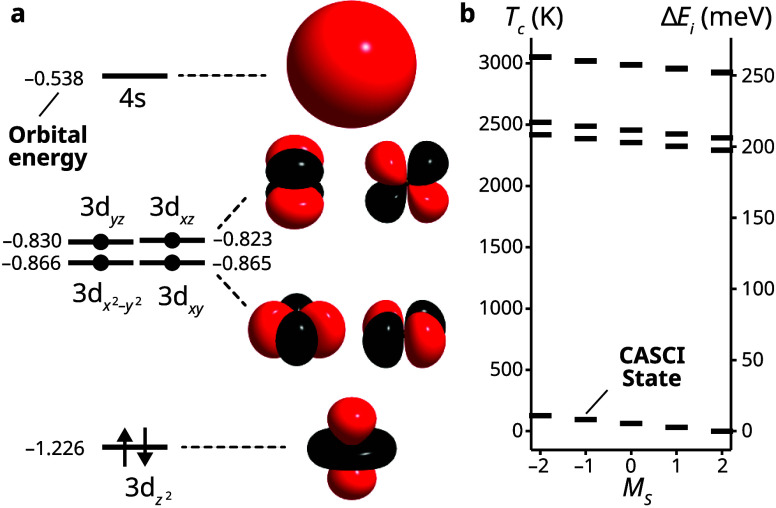
CASCI results for the Fe^2+^ cation using an active space containing six electrons in six orbitals. (a) Restricted open-shell Hartree–Fock (ROHF) orbitals used for the active space computations and a representative occupation pattern for the reference state. Orbital energies are shown in the absence of a magnetic field. The 3d orbitals obtained from ROHF are nondegenerate due to the lack of spherical symmetry (the 3d_*z* ^2^_ orbital being doubly occupied). (b) CASCI energy of the quintet states that arise from the atomic ^5^D term. The highest two quintets in the plot are nearly degenerate, causing them to overlap at this energy scale. Orbital visualization was performed using the VMD software package.^71^

**Table 1:** References Used for the Fe^2+^ Cation MORE-/HOT-ADAPT-VQE Calculations

reference	determinant	reference	determinant
0	$|3d_{z^{2}}^{2}3d_{x^{2}-y^{2}}^{\alpha }3d_{xy}^{\alpha }3d_{yz}^{\alpha }3d_{xz}^{\alpha }\rangle$	8	$|3d_{z^{2}}^{2}3d_{x^{2}-y^{2}}^{\alpha }3d_{xy}^{\alpha }3d_{yz}^{\alpha }3d_{xz}^{\beta }\rangle$
1	$|3d_{z^{2}}^{2}3d_{x^{2}-y^{2}}^{\alpha }3d_{xy}^{\alpha }3d_{yz}^{\beta }3d_{xz}^{\alpha }\rangle$	9	$|3d_{z^{2}}^{2}3d_{x^{2}-y^{2}}^{\alpha }3d_{xy}^{\alpha }3d_{yz}^{\beta }3d_{xz}^{\beta }\rangle$
2	$|3d_{z^{2}}^{2}3d_{x^{2}-y^{2}}^{\alpha }3d_{xy}^{\beta }3d_{yz}^{\alpha }3d_{xz}^{\alpha }\rangle$	10	$|3d_{z^{2}}^{2}3d_{x^{2}-y^{2}}^{\alpha }3d_{xy}^{\beta }3d_{yz}^{\alpha }3d_{xz}^{\beta }\rangle$
3	$|3d_{z^{2}}^{2}3d_{x^{2}-y^{2}}^{\alpha }3d_{xy}^{\beta }3d_{yz}^{\beta }3d_{xz}^{\alpha }\rangle$	11	$|3d_{z^{2}}^{2}3d_{x^{2}-y^{2}}^{\alpha }3d_{xy}^{\beta }3d_{yz}^{\beta }3d_{xz}^{\beta }\rangle$
4	$|3d_{z^{2}}^{2}3d_{x^{2}-y^{2}}^{\beta }3d_{xy}^{\alpha }3d_{yz}^{\alpha }3d_{xz}^{\alpha }\rangle$	12	$|3d_{z^{2}}^{2}3d_{x^{2}-y^{2}}^{\beta }3d_{xy}^{\alpha }3d_{yz}^{\alpha }3d_{xz}^{\beta }\rangle$
5	$|3d_{z^{2}}^{2}3d_{x^{2}-y^{2}}^{\beta }3d_{xy}^{\alpha }3d_{yz}^{\beta }3d_{xz}^{\alpha }\rangle$	13	$|3d_{z^{2}}^{2}3d_{x^{2}-y^{2}}^{\beta }3d_{xy}^{\alpha }3d_{yz}^{\beta }3d_{xz}^{\beta }\rangle$
6	$|3d_{z^{2}}^{2}3d_{x^{2}-y^{2}}^{\beta }3d_{xy}^{\beta }3d_{yz}^{\alpha }3d_{xz}^{\alpha }\rangle$	14	$|3d_{z^{2}}^{2}3d_{x^{2}-y^{2}}^{\beta }3d_{xy}^{\beta }3d_{yz}^{\alpha }3d_{xz}^{\beta }\rangle$
7	$|3d_{z^{2}}^{2}3d_{x^{2}-y^{2}}^{\beta }3d_{xy}^{\beta }3d_{yz}^{\beta }3d_{xz}^{\alpha }\rangle$	15	$|3d_{z^{2}}^{2}3d_{x^{2}-y^{2}}^{\beta }3d_{xy}^{\beta }3d_{yz}^{\beta }3d_{xz}^{\beta }\rangle$

**Figure 3:**
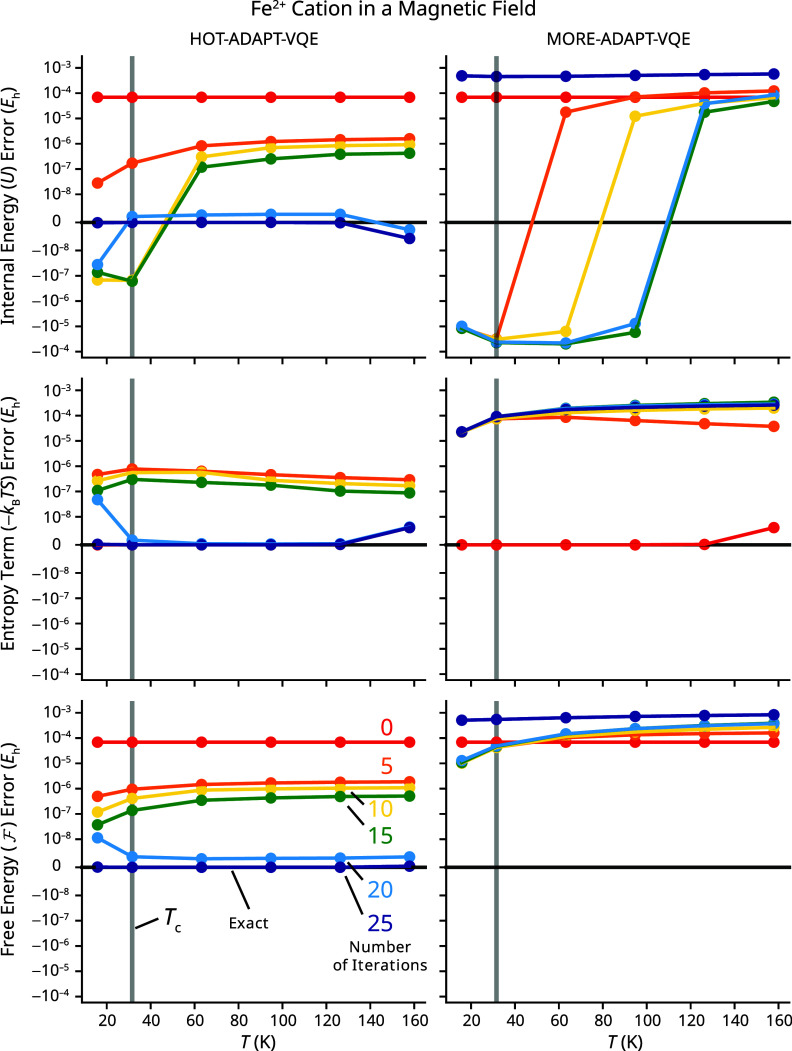
Plots of the error of energetic values from HOT-ADAPT-VQE (left) and MORE-ADAPT-VQE (right) at different iterations as a function of temperature for Fe^2+^ in a magnetic field. The *x*-axis is the temperature used for the two algorithms. From top to bottom, errors are reported in the internal energy *U*, the entropy term −*k*_B_ *TS*, and the free energy F . The different line colors correspond to different ADAPT-VQE iterations. The iteration 0 and 25-parameter values of the entropy contribution overlap for HOT-ADAPT-VQE, making the red curve difficult to see. Note that the red curve will be the same for both HOT- and MORE-ADAPT-VQE since no operators have been chosen, and the energies in the Boltzmann distribution come from diagonalizing *Ĥ* within the same set of references.

We begin our discussion of Figure 3 by analyzing the performance of HOT-ADAPT-VQE. For this problem, at iteration 0, HOT- and MORE-ADAPT-VQE yield the same energies and the errors are below chemical accuracy (1 m*E*_h_). Therefore, we focus on the relative improvements brought by the unitary. As expected, the free energy converges monotonically with each iteration for any given temperature. Conversely, the internal energy and entropy contributions to the free energy do not improve monotonically, as HOT-ADAPT-VQE only tries to minimize their sum. At all temperatures, HOT-ADAPT-VQE is eventually unable to add additional operators after the ansatz includes 25 generators. This is the case except for *T* = 2*T*_c_, where an additional operator leads to a negligible decrease in the free energy. While premature convergence to a local minimum is possible, this problem is more pronounced at low temperatures, where the algorithm prematurely ignores important states with low weights in the early stages of the simulation. In this situation, it is more likely that the small error comes from the increasing population of the first excited quintet states in the CASCI solution at higher temperatures. For example, because the references used include only one *M*_S_ = 2 determinant, the lowest-lying state of the first excited quintet cannot be prepared with the *M*_S_-conserving GSD operators. This is consistent with the fact that the internal energy and entropy values are both slightly below the CASCI values, suggesting that there are not enough states in the manifold to obtain the exact entropy, attributing a higher weight to the lower-energy states.

Compared with finite-temperature computations based on MORE-ADAPT-VQE states, HOT-ADAPT-VQE yields a significant improvement in the number of operators required to obtain accurate free energies. This is consistent with the fact that the MORE-ADAPT-VQE energy functional does not prioritize the low-lying eigenstates of *Ĥ*, leading to the selection of a sequence of unitaries that is not optimal to describe a finite-temperature state. This is illustrated quite dramatically in the fact that the internal energy, entropy, and free energy all become less accurate at 25 iterations of MORE-ADAPT-VQE than at 0 iterations. This is possible because MORE-ADAPT-VQE seeks to improve the unweighted average of the energies, which can cause the low-lying energies to increase as long as the high-lying energies decrease. This effect is shown in Figure 4, where a comparison of MORE-ADAPT-VQE at 0 and 25 iterations shows that the high-lying states (6–16) with low thermal populations improve at the expense of the important states (1–5). When the thermal ensemble is prepared, the high-lying energies will still be too high in energy to be significantly populated, but some of the low-lying energies will be much worse than they were at iteration 0. It is only at infinite temperature that equally weighted MORE-ADAPT-VQE is guaranteed to monotonically improve the internal energy *U* with each iteration. Given that the errors are greater than those of HOT-ADAPT-VQE, negative errors in *U* cannot be dismissed for MORE-ADAPT-VQE as coming from an insufficient number of references. It is possible, however, to obtain negative errors in *U* for MORE-ADAPT-VQE, despite the algorithm minimizing the unweighted average of individual state energies for a similar reason. If the relative energies between states are not accurately predicted, then the Boltzmann distribution will disproportionately populate states that are lower in energy, decreasing *U* and increasing *S* for the thermal ensemble, a phenomenon observed here. Variationality of the internal and free energies is guaranteed for both methods at *T* = 0.

**Figure 4:**
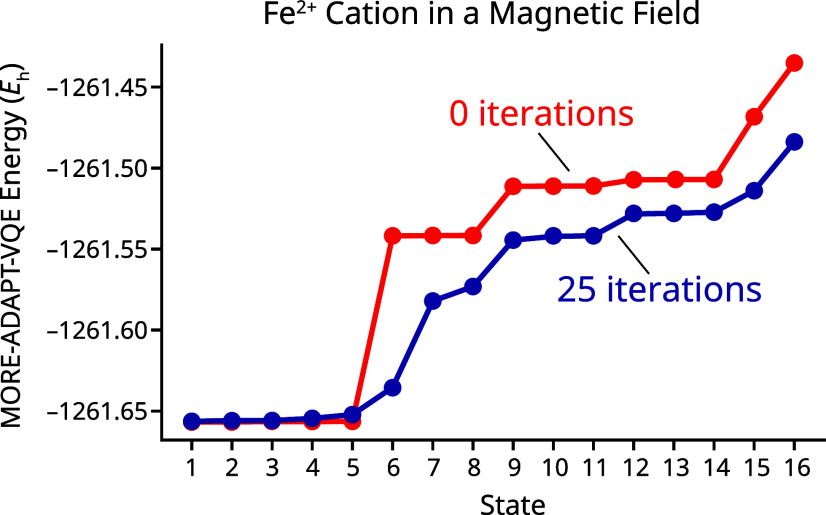
Plots of the energy of the MORE-ADAPT-VQE states for Fe^2+^ in a magnetic field at 0 and 25 iterations.

The performance of MORE-ADAPT-VQE could be improved by introducing unequal weights to accelerate convergence or by using fewer references, albeit at the cost of reduced accuracy at convergence. Both of these strategies, however, would mean taking advantage of knowledge about the true Gibbs state, information that is not typically available a priori. Based on the iron cation results, HOT-ADAPT-VQE seems to be extremely promising for preparing approximate thermal ensembles at low temperatures. In addition to the choice of weights, HOT-ADAPT-VQE removes the ambiguity of whether to spin-adapt the references. This is because HOT-ADAPT-VQE can break or restore total spin symmetry during the diagonalization of *H̅*, ensuring that the best multideterminantal basis is always used.

### 4.2 Cuprate Model

After examining the Fe^2+^ cation, we next applied HOT-ADAPT-VQE to a more challenging chemical system. A major potential application of finite-temperature methods is the study of electronic phase transitions, particularly in superconducting materials. An interesting example is the cuprate family, a class of high-temperature superconductors discovered in the late 1980s.^72,73^ Unlike conventional superconductors, the behavior of cuprates cannot be adequately described by the standard Bardeen–Cooper–Schrieffer (BCS)^74^ theory of superconductivity.^75^ These unconventional superconducting properties and a strong superexchange mechanism in the CuO_2_ planes^76^ make these materials interesting from an electronic structure point of view. Characterizing the associated antiferromagnetic interaction represents a challenging problem for quantum chemistry methods, due to strong correlation and the need for a large active space.^77,78^ However, recent works have extended ab initio quantum chemistry methods to compute the properties of cuprates, shedding light on some properties of these materials not captured by effective low-energy models.^79–81^

As a representative starting point, we consider a [Cu_2_O_7_]^10–^ planar fragment of D_2h_ symmetry, pictured in Figure 5. This fragment models the essential local electronic structure within the CuO_2_ planes of La_2_CuO_4_, a classic parent material for high-temperature cuprate superconductors.^82^ In the absence of doping, cuprate parent materials like La_2_CuO_4_ are Mott insulators;^83^ i.e., conventional band-theory approaches incorrectly predict metallic behavior. Indeed, when modeling this system, we identify significant challenges for variational finite-temperature quantum algorithms when the reference states (the starting point for HOT-ADAPT-VQE) do not appropriately capture correlation effects. Specifically, the absence of references that contribute to the correlated singlet ground state leads to incorrectly biasing the optimization toward a triplet state. This qualitative discrepancy underscores one of the challenges of accurately capturing the physics of cuprates and motivates our choice of the [Cu_2_O_7_]^10–^ fragment as a compelling benchmark test for quantum algorithms.

**Figure 5:**
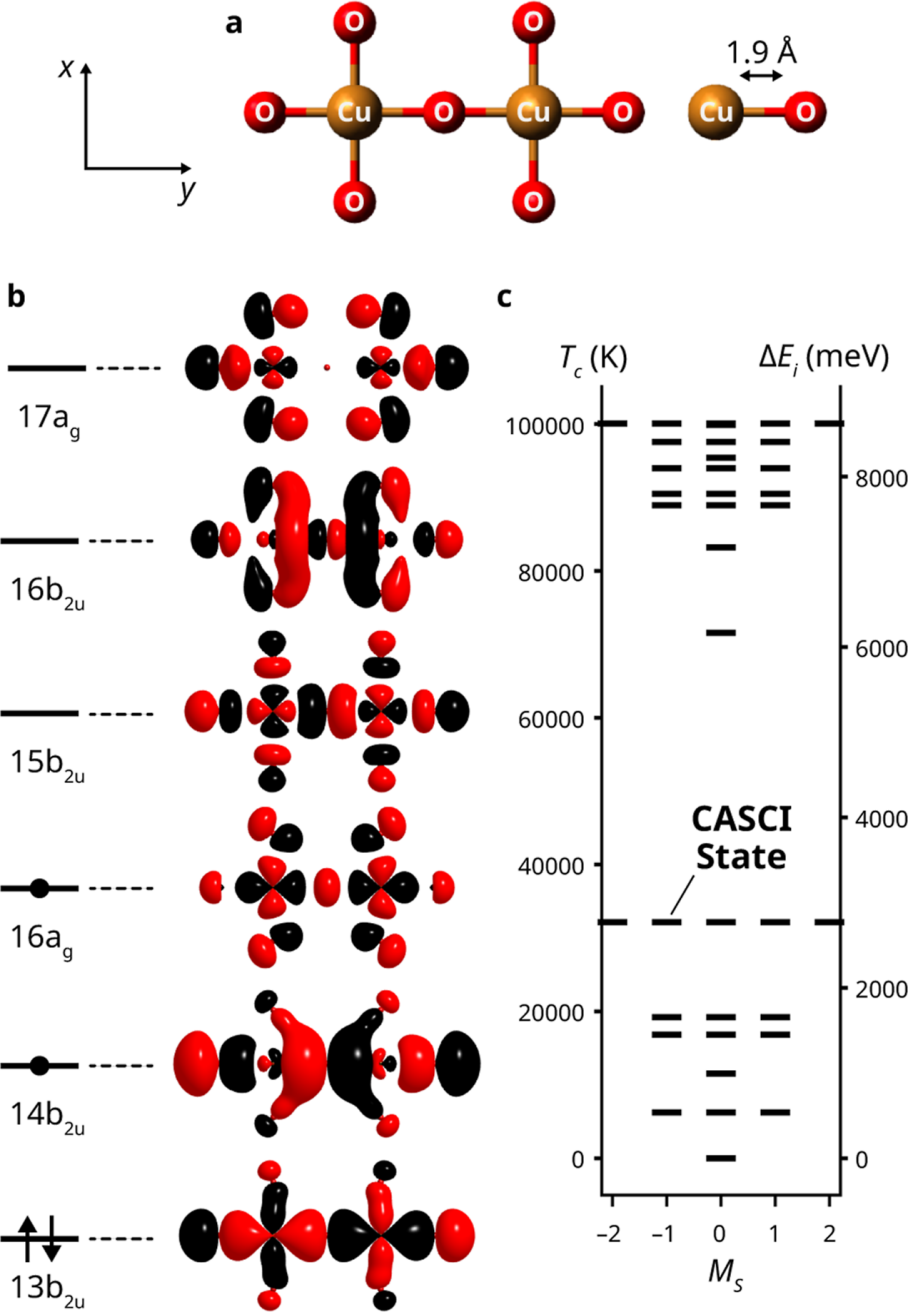
CASCI results for the [Cu_2_O_7_]^10–^ anion using an active space containing four electrons in six orbitals. (a) Geometry of the planar [Cu_2_O_7_]^10–^ anion studied in this work. All Cu–O distances are fixed to 1.90 Å. (b) State-specific CASSCF orbitals used for the active space computations and a representative occupation pattern for the leading determinants in the ground state. Orbital visualization was performed using the VMD software package.^71^ (c) The lowest 37 CASCI energies of the system.

In our [Cu_2_O_7_]^10–^ model, all Cu–O bond lengths were set to 1.90 Å, the experimental value for La_2_CuO_4_.^84^ An initial set of molecular orbitals was obtained by performing an RHF calculation in the cc-pVDZ basis.^69,70,85^ In designing our active space, we sought to include the ${3d}_{x^{2}-y^{2}}$ orbitals for each copper atom and the 2p_*y*_ orbital of the central oxygen atom, the minimum necessary active space to partially describe the superexchange mechanism. To this end, we performed state-specific CASSCF computations with a (4e,6o) active space, with starting orbitals selected to include two a_g_ and four b_2u_ orbitals, to account for orbitals that arise as a linear combination of the aforementioned atomic orbitals. CASSCF was used to target the lowest-energy ^1^B_2u_ singlet state, with the resulting orbitals used in the quantum algorithm studies. It is important to note that due to the several simplifications made in our model (minimal fragment model, absence of counterions, etc.), this choice of active orbitals does not lead to the lowest-energy CASSCF solution of the [Cu_2_O_7_]^10–^ fragment.

The resulting CASSCF model space reasonably describes the superexchange mechanism, as our antiferromagnetic *J* is quite accurate relative to experiments (138 meV from our model vs 120–160 meV for experiment).^76,86–88^ *J* was estimated according to the singlet–triplet case of the Landé interval rule^89^ as

37 $$J=\frac{E\left(T_{1}\right)-E\left(S_{0}\right)}{4}$$

where *E*(*S*_0_) and *E*(*T*_1_) are the energies of the lowest singlet and triplet states, respectively.

The closed-shell Aufbau determinant |13b_2u_^2^14b_2u_^2^⟩ and the set of singly excited determinants were used as the reference manifold, giving a total space of dimension 33 for HOT- and MORE-ADAPT-VQE. The low-temperature thermodynamic character of this system is primarily defined by the singlet–triplet gap between the lowest CASCI states in Figure 5. The associated critical temperature for this gap in our model system is 6243 *K* (538.0 meV). We report the energetics of [Cu_2_O_7_]^10–^ with different methods for temperatures ranging from 0.2 to 2 *T*_c_ in Figure 6. Our results include data for up to 250 iterations of each algorithm due to computational time limitations.

**Figure 6:**
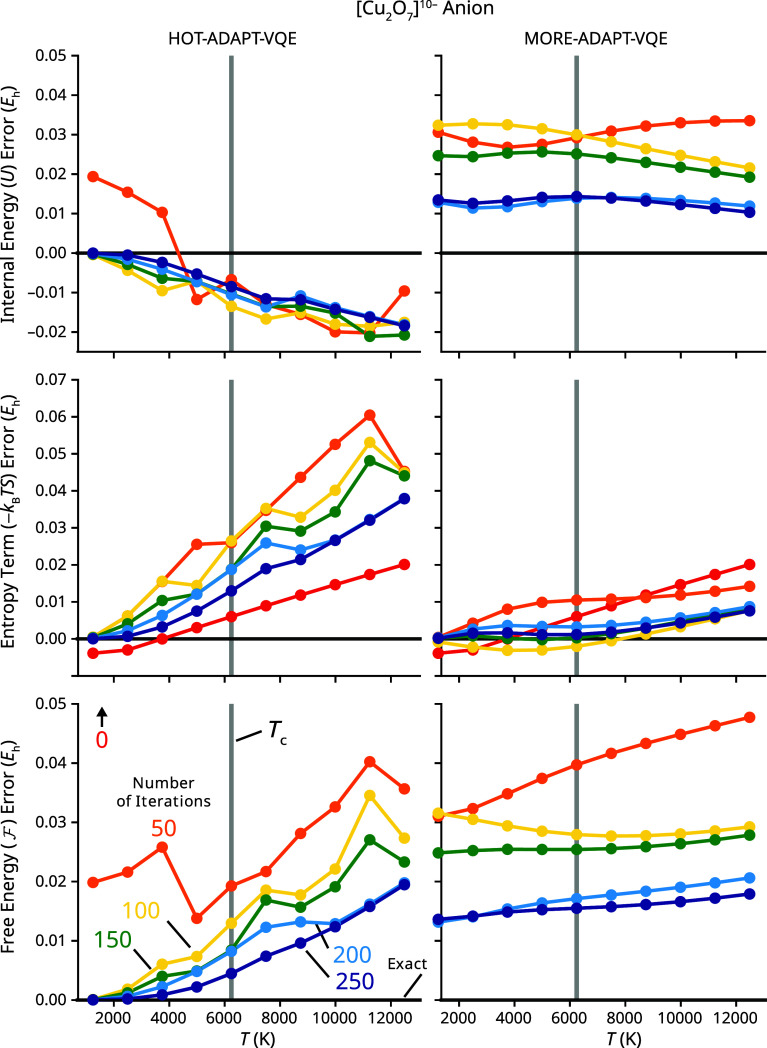
Plots of the error of energetic values from HOT-ADAPT-VQE (left) and MORE-ADAPT-VQE (right) at different iterations as a function of temperature for the [Cu_2_O_7_]^10–^ model. Conventions are the same as in Figure 3 unless otherwise specified. The internal energy and free energy errors lie outside the range of the graph (≈0.2 *E*_h_) at iteration 0 but do not depend on the method used. HOT-ADAPT-VQE data points for *T* equal to 0.2 and 0.4 *T*_c_ were obtained by interpolation from the 0.6 *T*_c_ solutions.

In our computations, we observe that for all temperatures, HOT-ADAPT-VQE eventually attempts to add the same operator twice in a row due to a failure to optimize it in the previous macroiteration, despite using a very tight convergence criterion in the BFGS algorithm. If the last operator in the ansatz has a parameter gradient that is too small for BFGS to continue, adding the same operator will just add another parameter with the same gradient to the end of the ansatz, making it pointless to continue the algorithm, as is the case with traditional ADAPT-VQE. We define this situation as premature convergence if the individual states ${\left\{|\Psi_{i}\rangle \right\}}_{i=1}^{k}$ do not have energy equal to the *k* lowest energies obtained with CASCI (only for symmetries accessible through the action of *Û*(**θ**) on the references ${\left\{|\phi_{i}\rangle \right\}}_{i=1}^{k}$ ). We were generally able, however, to overcome this premature convergence by reinitializing all the ansatz parameters to 0 (as opposed to using parameters from a previous ADAPT-VQE iteration as a guess) and performing a new HOT-VQE subroutine with the same ansatz. Using the resulting reoptimized parameters, HOT-ADAPT-VQE was allowed to proceed. Additional reboots of the algorithm were performed whenever the algorithm attempted to add the same operator twice consecutively. This technique was used to obtain the data reported in Figure 6. To illustrate this rebooting behavior, we include the iteration-by-iteration convergence of F at the critical temperature (*T* = *T*_c_) in Figure 7. Rebooting was not used for MORE-ADAPT-VQE because it did not suffer from premature convergence to a local minimum in F .

**Figure 7:**
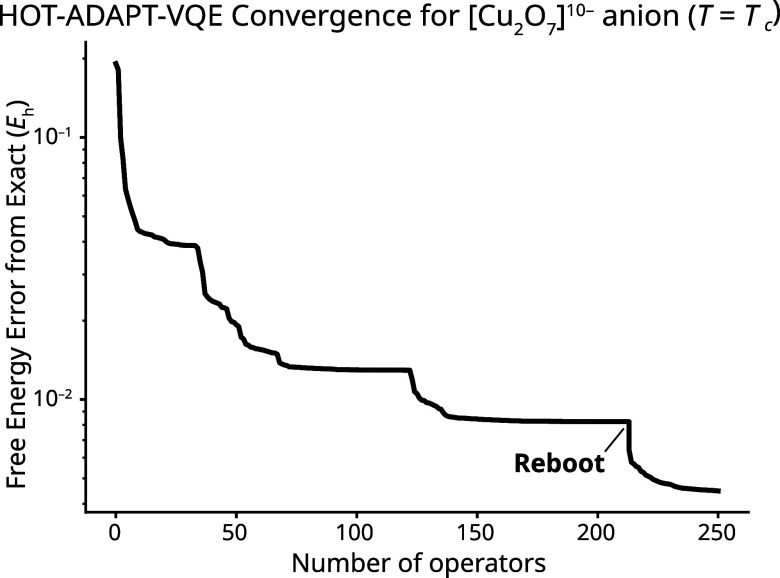
Plot showing the convergence of the free energy of [Cu_2_O_7_]^10–^ using HOT-ADAPT-VQE with *T* = *T*_c_. The large drop at iteration 213 corresponds to the first reboot of the algorithm, where the line connects the prematurely converged energy and the energy after reoptimizing the parameters for the same ansatz.

The fact that reinitializing the parameters to 0 can immediately improve the energy is somewhat surprising based on previous reports in the literature. In ref 63, the authors report trying a large number of random initializations to escape a TEPID-ADAPT-VQE solution that failed to include a low-lying excited state for a system, capturing a higher-lying one instead. Using these random initializations did not improve the energy for the same ansatz. We defer a more involved comparison of the energy landscapes from the two methods to future work, but we note that this behavior of HOT-ADAPT-VQE is consistent with that of the traditional ADAPT-VQE ansatz.^90^

Unfortunately, even with our rebooting procedure, HOT-ADAPT-VQE prematurely converged at 0.2 and 0.4 *T*_c_, essentially collapsing to form the lowest-lying triplet. This is particularly troubling, given that traditional ADAPT-VQE could at least obtain the correct ground state of the system. We tried performing such a traditional ADAPT-VQE calculation until convergence to the ground state singlet (*E*_ADAPT_ – *E*_CASCI_ < 10^–8^ *E*_h_) to obtain an initial ansatz *Û*(**θ**) which had 111 operators. Using this unitary, a reboot was performed by reinitializing all of the parameters to 0 and switching to the HOT-ADAPT-VQE algorithm, using the same references as before. HOT-ADAPT-VQE was only allowed to perform 20 total reboots due to the long time required to reinitialize the parameters repeatedly. Even with these reboots, at 0.2 *T*_c_, HOT-ADAPT-VQE still had negligible contributions in the ensemble from states other than the lowest-lying triplet, despite beginning with the correct operator sequence associated with the exact ground state. Similarly, we also find that converging an ADAPT-VQE calculation and then switching to the HOT-ADAPT-VQE algorithm without reboots does not overcome issues of premature convergence.

This failure shows the degree of difficulty in treating a low-temperature system with poor initial guesses of the eigenstates of *Ĥ*. In our model, the multideterminantal character of the singlet ground state is not captured well by the set of reference states, leading to a reversal of the predicted energy ordering, with the triplet state being incorrectly predicted to lie 0.802 eV lower in energy than the singlet. Consequently, the Boltzmann probabilities are inverted and HOT-ADAPT-VQE favors adding operators that primarily lower the energy of the triplet state. We ultimately used the interpolation strategy from ref 63 to obtain estimates for the Gibbs states at temperatures equal to 0.2 and 0.4 *T*_c_. For a given HOT-ADAPT-VQE iteration, we report 0.2 and 0.4 *T*_c_ based on the same *Û*(**θ**) and **C** obtained with HOT-ADAPT-VQE for *T* equal to 0.6 *T*_c_. The only difference from the 0.6 *T*_c_ ensemble is the set of ensemble weights {*p*_*i*_}, which are obtained with the Boltzmann distributions for temperatures of 0.2 and 0.4 *T*_c_. It is important to note that while HOT-ADAPT-VQE has convergence problems, it is typically better than MORE-ADAPT-VQE at obtaining accurate free energies with the same number of operators, accomplishing the original goal of this work. It is notable that for this system, MORE-ADAPT-VQE maintains a significantly lower error in the entropy, despite having a higher error in the free energy. The low error in the entropy, combined with the high error in the internal energy, is due to the nature of MORE-ADAPT-VQE. In attempting to optimize the unweighted average of the energies, MORE-ADAPT-VQE can produce states that are correctly spaced in energy but have larger absolute energy errors.

Figure 6 clearly shows that HOT-ADAPT-VQE outperforms MORE-ADAPT-VQE in predicting free energies at lower temperatures. To quantitatively compare these two methods on equal footing in terms of ansatz length, in Table 2, we show the errors in the free energy F obtained after 250 iterations of both algorithms. The data in Table 2 show that HOT-ADAPT-VQE is generally more accurate, with its advantage diminishing as the temperature increases, eventually yielding a slightly less accurate free energy than MORE-ADAPT-VQE at *T* = 2 *T*_c_. This decline in accuracy is unsurprising, as in the limit of *T* → ∞, the Boltzmann distribution will be uniform for all eigenstates, removing any freedom in the ensemble weights {*p*_*i*_} and making MORE-ADAPT-VQE and HOT-ADAPT-VQE equivalent. Additionally, in this high-temperature limit, the coupling between references {|ϕ_*i*_⟩} through **H̅** (see eq 13) is also irrelevant since the internal energy simplifies to the unweighted trace of 1/kH̅, a quantity invariant under rotations among the references. We also remind the reader that the measurement requirements of HOT-ADAPT-VQE will be much greater than those for a MORE-ADAPT-VQE ansatz of the same length, so that if coherence time is not the primary problem for the hardware being used, this improved accuracy may be less meaningful. However, within the chemically relevant and challenging low-temperature regime, HOT-ADAPT-VQE demonstrates substantial promise due to its direct optimization of the Helmholtz free energy.

**Table 2:** Errors (in *E*_h_) Relative to the Exact Free Energies of the [Cu_2_O_7_]^10–^ Fragment at 250 Iterations for HOT- and MORE-ADAPT-VQE

*T*/*T*_c_	HOT-ADAPT-VQE	MORE-ADAPT-VQE
0.2	2.847 × 10^–5^	1.362 × 10^–2^
0.4	1.844 × 10^–4^	1.416 × 10^–2^
0.6	8.724 × 10^–4^	1.486 × 10^–2^
0.8	2.204 × 10^–3^	1.525 × 10^–2^
1.0	4.477 × 10^–3^	1.550 × 10^–2^
1.2	7.399 × 10^–3^	1.576 × 10^–2^
1.4	9.617 × 10^–3^	1.612 × 10^–2^
1.6	1.237 × 10^–2^	1.660 × 10^–2^
1.8	1.579 × 10^–2^	1.720 × 10^–2^
2.0	1.947 × 10^–2^	1.790 × 10^–2^

## 5 Conclusions

In this work, we introduced HOT-ADAPT-VQE, a novel hybrid quantum-classical algorithm designed to adaptively construct Gibbs states by explicitly minimizing the Helmholtz free energy through continuous mixing and optimization of an ensemble of reference states. Compared with a simpler strategy based on equally weighting each reference state (MORE-ADAPT-VQE), HOT-ADAPT-VQE can often accurately determine the free energy using significantly shallower operator products. This improved efficiency is achieved by actively adapting the ensemble weights *p*_*i*_ based on current state energies, thereby focusing the ansatz expressiveness on the optimization of those states that dominate the thermal ensemble. Despite these promising features, we have observed some troubling pathologies with variational thermal state algorithms. Specifically, HOT-ADAPT-VQE may fail to converge to the global minimum of F when important states have very low initial populations and thus they are not improved in the optimization process. This challenge is illustrated in our cuprate model system, where the strongly correlated singlet ground state is poorly represented by the initial set of reference determinants. In many situations, rebooting the algorithm by reinitializing the ansatz parameters to zero can allow the algorithm to continue, though even this approach can fail for sufficiently low temperatures. It is encouraging to observe that interpolation from higher temperatures^63^ provides a simple and effective solution to the problem of premature convergence, enhancing the practical utility of HOT-ADAPT-VQE. A more thorough exploration of the numerical parameter optimization details would be beneficial going forward, including the choice of the optimizer itself.

The choice of reference determinants is an open problem, in terms of both the number of references needed and the selection of references for any given value of *k*. In the case of our cuprate model, CASSCF was used to identify a classically tractable active space; then, a subset of the determinants in that subspace was used. In a real use case where larger active spaces could be simulated on quantum hardware, CASSCF could still be useful for identifying a small set of important determinants to use as references. To obtain the same number of references, the CASSCF active space would not need to grow larger with system size. As mentioned before, situations where thermal effects are localized could have constant requirements for *k* with the system size, which would make CASSCF reference selection viable. In any case, the results of HOT-ADAPT-VQE are extremely sensitive to the choice of references, making this a key area of future work.

A natural direction for future work is to generalize HOT-ADAPT-VQE to the grand canonical ensemble, relevant for systems exchanging electrons with their environment. In the grand canonical ensemble, the electron number is not fixed but controlled by the chemical potential μ, and the corresponding free energy functional is given by

38 Ω[ρ̂]=U−TS−μN

where *N* is the average number of electrons, expressible as Tr(ρ̂N̂) , where *N̂* is the number operator. Extending HOT-ADAPT-VQE to this ensemble requires generalizing the selection of reference states (which will span different sectors of the Fock space) and considering nonparticle-conserving generalizations of the ADAPT-VQE operator pool. Another desirable extension of HOT-ADAPT-VQE is to model Hamiltonians describing Fermions coupled to phonons. In real finite-temperature chemical and solid-state systems, vibrational modes (phonons) often possess energies comparable to or lower than electronic excitation energies, resulting in significant coupling of these two degrees of freedom that profoundly affects thermal properties. Additionally, the flexibility in choosing the operator pool and reference states within HOT-ADAPT-VQE offers opportunities for future algorithmic improvements. In summary, the good performance demonstrated by HOT-ADAPT-VQE, combined with clear avenues for further generalization and optimization, makes it a promising quantum algorithm for accurate and practical thermal state calculations on quantum computers. Note added in proof: while finalizing this work, we became aware of another quantum algorithm for thermal simulation (see ref 91).
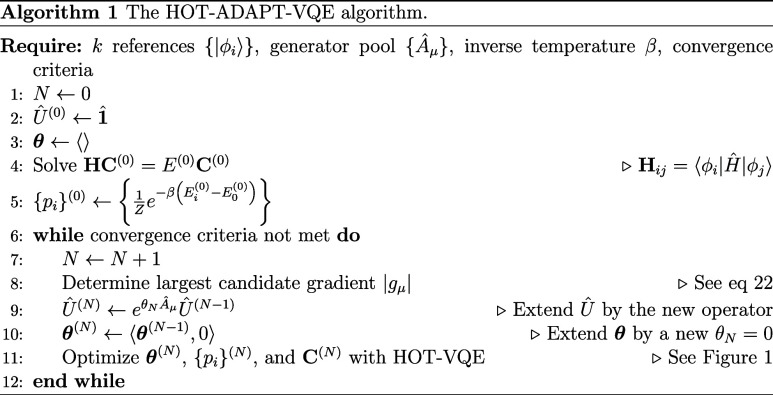


## Supplementary Material





## Data Availability

Code used in this project is available in a local fork of the QForte package at https://github.com/hgrimsl/qforte/tree/new_thermal and in the Forte package at https://github.com/evangelistalab/forte/tree/qforte_stuff. All data are available upon reasonable request.

## Notes

(TXT)
Output from the Forte package for Fe^2+^ containing electron integrals, orbital symmetries, etc.; output from the Forte package for [Cu_2_O_7_]^10–^ containing electron integrals, orbital symmetries, etc.; thermal properties of Fe^2+^ computed using CASCI; and thermal properties of [Cu_2_O_7_]^10–^ computed using CASCI (ZIP)
